# Treatment of laundry wastewater by solar photo-Fenton process at pilot plant scale

**DOI:** 10.1007/s11356-020-11151-x

**Published:** 2020-10-16

**Authors:** Ana Belén Esteban García, Kacper Szymański, Sylwia Mozia, José Antonio Sánchez Pérez

**Affiliations:** 1 Solar Energy Research Centre (CIESOL), Joint Centre University of Almería-CIEMAT, 04120 Almería, Spain; 2 Chemical Engineering Department, University of Almería, 04120 Almería, Spain; 3 Faculty of Chemical Technology and Engineering, Department of Inorganic Chemical Technology and Environment Engineering, West Pomeranian University of Technology in Szczecin, Pułaskiego 10, 70-322 Szczecin, Poland

**Keywords:** Advanced oxidation process, Surfactants, Sodium dodecyl sulphate, Mineralization, Compound parabolic collector

## Abstract

Laundry sector consumes a huge amount of water which is usually discharged as wastewater instead of being reused. The application of biological treatment of laundry wastewater coupled with post-treatment utilizing advanced oxidation processes creates a possibility to recycle water to the washing process. However, the investigations on such systems are very limited. In the present work, a novel approach of post-treatment of laundry wastewater utilizing solar photo-Fenton operated at a pilot scale in a compound parabolic collector (CPC) photoreactor is proposed. Sodium dodecyl sulfate (SDS) was used as a representative of surfactants applied in the laundry system. The effect of feed matrix was investigated using distilled water as a reference matrix and synthetic wastewater simulating the composition of biologically pre-treated laundry wastewater. Different concentrations of H_2_O_2_ (50–400 mg/L) and ferrous iron (2.75–10 mg/L) were assayed. For comparison purpose, experiments at neutral pH using ethylenediamine-N,N′-disuccinic acid (EDDS) as an iron complexing agent were carried out. A high SDS removal efficiency was obtained under both neutral and acidic pH, reaching 89% and 96%, respectively, in just 8 min. However, the remaining organic load originating from EDDS needs application of further post-treatment steps. Therefore, the solar photo-Fenton operated under acidic pH was found to be a more promising approach of post-treatment of laundry wastewater aimed at its reuse.

## Introduction

Laundry wastewater is generated during the washing of clothes at household and industrial scale. Modern laundries use 10 L of water per 1 kg of dry clothing, while the water consumption in the old type laundries can be even two to three times higher. Actually, even 90% of the used water can be discharged as wastewater (Yaseen et al. 2019). The laundry wastewater is characterized mainly by a high content of surfactants commonly used in the washing process, high microbiological load, and a large amount of pollutants, primarily fats, dyes, oils, and suspended solids removed from the washed textile items (Braga and Varesche 2014). Such a very complex composition of laundry wastewater requires a special approach to its treatment and application of various techniques. The conventional wastewater treatment methods are usually not efficient enough in the case of laundry wastewater, which causes an environmental pollution problem. For this reason, the development of efficient and cost-effective treatment technology is very important (Patil et al. 2020).

One of the treatment methods used in wastewater treatment plants is a biological process. Biological treatment involves the metabolic activity of living organisms, such as bacteria and fungi, which are present in natural water and soil. The biological processes, however, not always provide satisfactory results, while some contaminants can be toxic for the microorganisms or just non-biodegradable. The presence of non-biodegradable compounds is observed in the case of laundry wastewater; therefore, before the biologically treated wastewater reaches the environment, it should be polished with other methods (Patil et al. 2020).

The advanced oxidation processes (AOPs) could be promising towards the degradation of persistent contaminants which are not removed during a biological process (Patil et al. 2020). The high efficiency of AOPs is associated with the generation of highly reactive HO^•^ radicals, which can oxidize many organic compounds (Pignatello et al. 2006). Eventually, complete mineralization to CO_2_, water, and mineral compounds can be obtained. The most extensively studied AOPs are photo-Fenton process (PF) (Ameta et al. 2018), photocatalysis (Amor et al. 2019), UV/H_2_O_2_ (Miklos et al. 2018), and others (Malato et al. 2009). However, all of these techniques have some disadvantages, mainly high costs due to the high electricity demand (UV lamps) or large amounts of photocatalysts, creating additionally separation problems (Klavarioti et al. 2009). The PF process is one of the most widely used AOPs for the wastewater treatment due to the ability to detoxify and disinfect wastewater streams containing persistent contaminants (Garrido-Cardenas et al. 2020). This technique is environmentally friendly and economically profitable (Umar et al. 2010; Yu et al. 2015); it does not require any catalyst and is relatively simple to perform. Additionally, in case of some recalcitrant pollutants the PF can more effective than other AOPs (Hansson et al. 2012). During the Fenton reaction which involves the combination of Fe^2+^ and H_2_O_2_ the HO^•^ radical is formed. The reaction rate is enhanced by utilizing UV radiation (photo-Fenton). In the presence of the light the iron ions are oxidized and reduced cyclically (Fe^3+^ can absorb light yielding hydroxyl radicals and simultaneously its reduction to initial Fe^2+^ takes place). The cost of implementing this technique to its commercial use can be greatly reduced by using solar energy as a UV light source (Miralles-Cuevas et al. 2017). From this point of view, the solar PF process can be an effective way to treat laundry wastewater (Yang and Wang 2009). The highly oxidative hydroxyl radicals will be the main species responsible for removal of persistent pollutants from the laundry effluents.

Sodium dodecyl sulphate (SDS) was selected as a model surfactant since it is a commonly applied component of many domestic and industrial detergents, personal hygiene, and cosmetic products, and its use gradually increases year-by-year (Adak et al. 2005). It is estimated that these days, SDS accounts for around 40% of all detergents consumption, creating a very serious problem in its efficient removal using current techniques. Moreover, this compound exhibits recalcitrant nature and can be harmful to human health and aquatic environment causing irreversible damage on the ecosystem (Malakootian et al. 2016; Collivignarellin et al. 2019). Harmfulness of SDS is primarily due to its accumulation in the environment and organisms, rather not its direct consumption, and cause long lasting effects (Bavcon Kralj et al. 2020; National Center for Biotechnology Information (n.d.) https://pubchem.ncbi.nlm.nih.gov/compound/Sodium-dodecyl-sulfate). Nevertheless, the consumption of this chemical could cause allergic reactions in human (National Center for Biotechnology Information (n.d.) https://pubchem.ncbi.nlm.nih.gov/compound/Sodium-dodecyl-sulfate).

In the subject literature, there are very limited reports on SDS removal in the photo-Fenton process. Some researchers studied the variations of PF for SDS removal in effluents from carwash (Rahimpour et al. 2020), industrial wastewater (Mirbahoush et al. 2019), textile wastewater (Mahamallik and Pal 2017), soft drink wastewater (Malakootian et al. 2016), and soil washing effluent (Bandala et al. 2013). However, to the best of our knowledge, reports on photo-Fenton process application to laundry wastewater post-treatment have not been published yet.

The main objective of the presented paper was to investigate the possibility of application of the solar photo-Fenton process for removal of the anionic surfactant sodium dodecyl sulphate from synthetic wastewater of a composition simulating the quality of laundry wastewater after biological treatment. The idea has arisen from our previous works on the treatment of laundry wastewater (Bering et al. 2018a, b; Mozia et al. 2016). During these investigations it was observed that surfactants are not removed efficiently enough during biological treatment and further polishing of the effluent is necessary. One promising approach is the application of AOP such as the solar photo-Fenton system. In the presented research, the effect of process parameters on the degradation efficiency as well as the quality of the product was evaluated. Furthermore, experiments at neutral pH using ethylenediamine-N,N’-disuccinic acid (EDDS) as an iron complexing agent were carried out.

## Materials and Methods

### Chemicals and aqueous matrices

Sodium dodecyl sulphate (SDS, NaC_12_H_25_SO_4_, 98.5% w/w), tetrabutylammonium bisulphate (97%, w/w), ethylenediamine-N,N′-disuccinic acid (EDDS, 35%, w/v), titanium(IV) oxysulphate (1.9%, w/v), ascorbic acid (99%, w/w), sodium bisulphite (40% w/v), and formic acid (95%, w/v) were supplied by Sigma-Aldrich. Chloroform (HPLC grade), and sulphuric acid (98%, w/v) were acquired from J.T. Baker. 1,10-phenantroline (99% w/w), acetic acid (99.7% w/v), hydrochloric acid (37% w/v), hydrogen peroxide (33% w/v), acetonitrile (HPLC grade), ferrous sulphate heptahydrated (FeSO_4_·7H_2_O), and ferric sulphate hydrate (75% w/w) were obtained from Panreac. Sodium formate (99%, w/w) was supplied by Merck Millipore (Darmstadt, Germany), while methylene blue (98%, w/w) was purchased from Chempur (Poland). Ultrapure water was produced with a Millipore Direct-Q® Ultrapure Water System (Bedford, MA, USA). The constituents for preparing synthetic laundry wastewater (SW) were bactopeptone (25 mg/L, BD-Difco) and meat extract (20 mg/L, Biolife); urea (30 mg/L), calcium chloride (140 mg/L), and dipotassium phosphate (5 mg/L) supplied by Panreac; and magnesium sulphate (50 mg/L) and ammonium sulphate (5 mg/L) obtained from Sigma Aldrich. The composition of the synthetic wastewater with reference to total organic carbon and inorganic salts content represented the quality of the real biologically treated laundry wastewater investigated previously (Bering et al. 2018a, b; Mozia et al. 2016).

### Experimental set-up

**Fig. 1:**
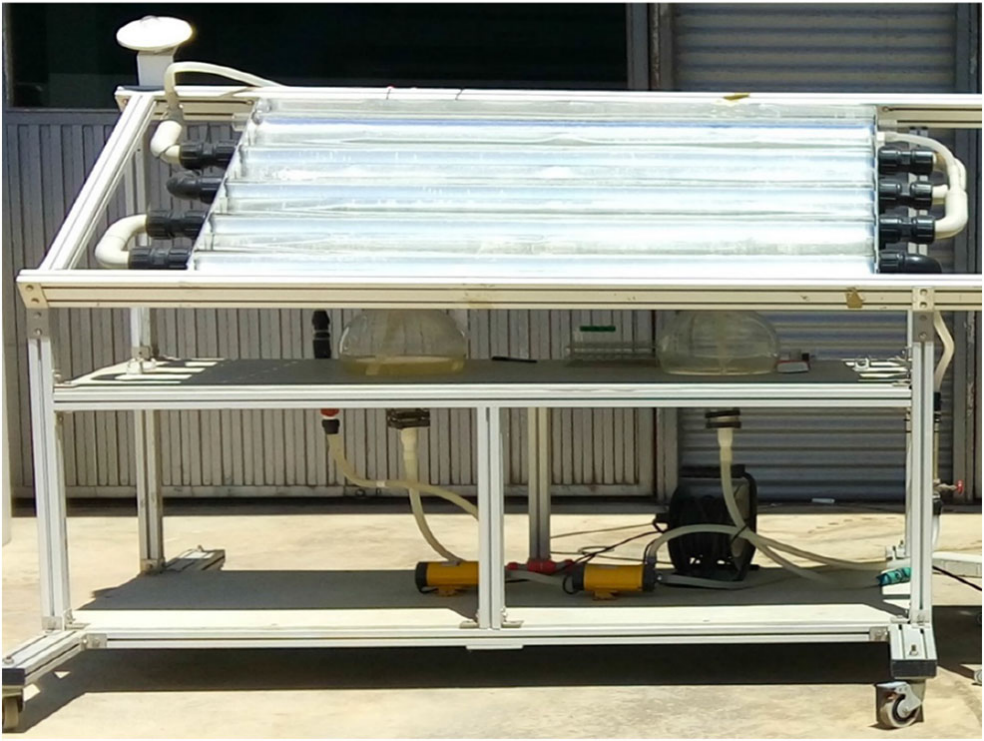
Pilot tubular photo-reactor equipped with a compound parabolic collector (CPC) situated in Solar Energy Research Center (CIESOL, University of Almería, Spain)

All PF assays were carried out at pilot plant scale in a tubular photo-reactor equipped with a compound parabolic collector (CPC; Fig. 1), under solar natural radiation in the Solar Energy Research Center (CIESOL, University of Almería, Spain). The solar plant consisted of two twin photo-reactors with borosilicate glass tubes (5-cm diameter) installed on a south-facing tilted platform at a local latitude of 37° (Cabrera Reina et al. 2018). This plant allows conducting two experiments simultaneously in the same conditions. The total illuminated surface of each photo-reactor was 0.42 m^2^, while the total (*V*_*T*_) and the illuminated volume (*V*_*i*_) were 9 L and 4.8 L, respectively. Assays were run in a batch mode, and the aqueous solutions were recirculated in the system through centrifugal pumps with a water flow rate of 22 L/min and a mixing time around 7 min. UVA radiation, temperature, and pH were monitored by a UV radiometer (Delta Ohm LP UVA 02 AV, spectral range from 327 to 384 nm), a temperature probe (Crison 6050), and a pH probe (Crison 5335), respectively. Data measured online were acquired by a data acquisition card (LabJack U12) connected to a computer. The solar experiments were performed at noon and the average value of the global UVA irradiance and temperature were 26 ± 2 W/m^2^ and 29.0 ± 0.8 °C, respectively.

In all assays, the pH was adjusted at 2.8 with sulphuric acid (1 M) and the solar exposition time was 120 min. Firstly, the glass tubes and twin photo-reactors were covered with black canvas and aluminium paper, respectively. Having done that, the two CPC tanks were loaded with the aqueous matrix and recirculated during 10 min for mixing. After that, hydrogen peroxide was added and mixed for 10 min. A control sample was collected for checking the initial oxidant concentration. Consecutively, the system was supplemented with ferrous iron and uncovered, starting the PF reaction. In case of the dark Fenton process the system remained covered during the whole experiment. Samples were directly taken from CPC reactor every 2 min during the first 10 min, every 5 min during the following 10 min, and later samples were collected every 10 min. Hydrogen peroxide, dissolved iron, and SDS concentrations were analysed in each sample. In addition, the mineralization degree of SDS was assessed in all samples.

Two experimental sets in outdoor conditions with two aqueous solutions (distilled water (DW) and synthetic wastewater (SW)) were conducted with 40 mg/L and 2 mg/L of SDS. The concentration of SDS, being a representative of anionic surfactants, was selected on a basis of the previous research on treatment of real laundry wastewater (Mozia et al. 2016; Bering et al. 2018a, b). Different concentrations of hydrogen peroxide (400, 200, 100, and 50 mg/L) and ferrous iron (10, 5.5, and 2.75 mg/L) were assayed. Fenton (F) experiments were carried out using the SDS concentration of 2 mg/L as a blank. Additionally, experiments at neutral pH were carried out with Fe^3+^:EDDS complex. The concentration of Fe^3+^ was 5.5 mg/L and the molar ratio of Fe^3+^:EDDS was 1:1.

### Analytical determinations

Before the quantification of hydrogen peroxide and ferrous iron concentrations, the samples were filtered through 0.2-μm nylon filters (Merck Millipore®). The absorbance of filtered samples with H_2_O_2_ and dissolved ferrous iron were determined at 410 nm with titanium(IV) oxysulfate reagent and 1,10 phenanthroline solution at 510 nm using standard methods, DIN 38 402 H15 (LOQ was 2.9 · 10^−2^ mM and SD was 4 · 10^−3^ mM) and ISO 6332 (LOQ was 4.5 · 10^−3^ mM and SD was 6.1 · 10^−4^ mM), respectively. The H_2_O_2_ and dissolved ferrous iron concentrations were obtained on the calibration curves. The concentration of sodium dodecyl sulphate was measured by colorimetric method adapted from (Koga et al. 1999) using methylene blue and chloroform. The detection limit of this method was 0.1 mg/L. The Fe^3+^-EDDS complex was determined by a 1200 Series system liquid chromatography with diode array detector (UHPLC-DAD) from Agilent Technologies (Waldbronn, Germany) according to Soriano-Molina et al. (2019). Dissolved organic carbon (DOC) and dissolved inorganic carbon (IC) were measured using filtered samples, with a Shimadzu-V CPH TOC analyser, which LOQ was 1 mg/L.

## Results and discussion

### Effect of iron concentration on SDS removal

**Fig. 2:**
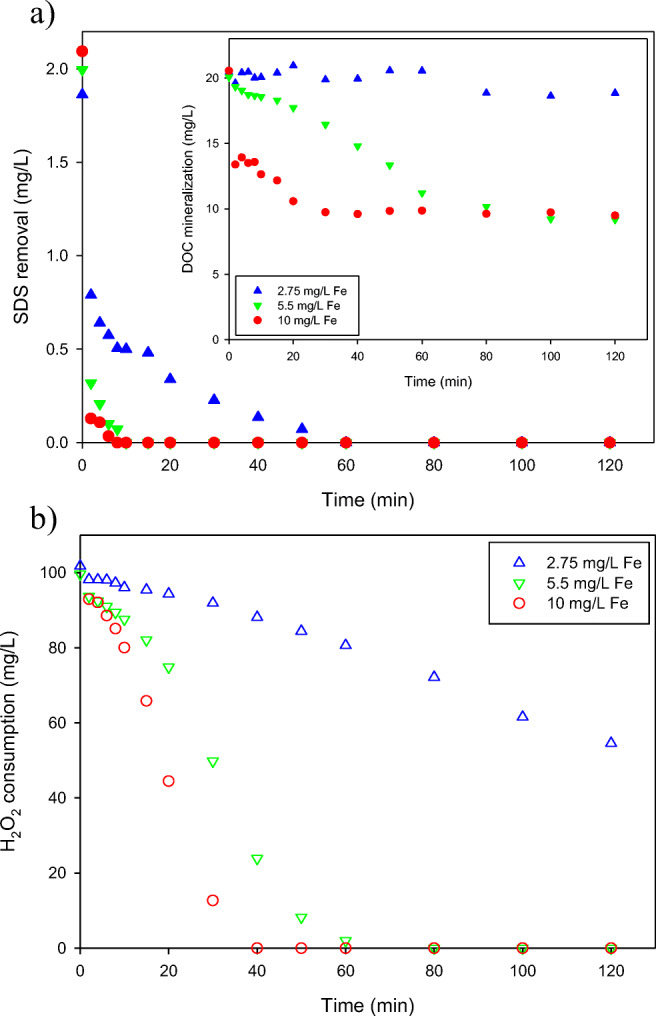
Effect of iron concentration (10, 5.5, and 2.75 mg/L) with 100 mg/L H_2_O_2_ in synthetic secondary effluent (SW) during solar PF process on **a** SDS removal and DOC mineralization (inserted figure) of 2 mg SDS/L. **b** Consumption of hydrogen peroxide

The first experimental set was designed in order to select the most effective iron concentration to remove and mineralize 2 mg SDS/L in a synthetic wastewater by solar photo-Fenton at acidic pH (2.8) using a CPC reactor. Results are shown in Fig. 2. In general, at higher iron content the process proceeds with higher efficiency due to (i) a faster regeneration of Fe^2+^ which leads to its better availability for hydroxyl radical production from H_2_O_2_ and (ii) a better utilization of H_2_O_2_, which is mainly consumed in the reaction of HO^•^ formation, not in side reactions. Furthermore, at higher iron concentration, the amount of H_2_O_2_ required for the same degradation degree is lower (Zapata et al. 2009). Concerning SDS removal, Fig. 2a shows an initial drop by Fenton process in the first few minutes (2 min) due to the addition of the catalyst in the form of ferrous iron following Reaction (1). This effect produces around 58%, 84%, and 94% of SDS removal for 2.75 mg Fe/L, 5.5 mg/L, and 10 mg/L of ferrous iron added, respectively. This means the Fenton effect is rather important in pollutant removal only if high iron concentrations are used. After this first reaction stage, ferric iron generated in Reaction (1) is reduced via solar radiation (Reaction (2)) to ferrous iron, thus closing the redox iron cycle and the degradation becomes progressive. Reaction (1) is independent of UV-light and only depends on iron and hydrogen peroxide concentrations. After the initial degradation, SDS profile followed a pseudo-first order rate although could not be appreciated, since its degradation was very fast. After 10 min of the photo-Fenton treatment, more than 98% of SDS was removed with the highest concentration of ferrous iron (10 mg/L), and for 5.5 mg/L was equal to 97%. These results demonstrated that increasing iron concentration did not produce a substantial increase in the degradation rate of SDS. Nevertheless, 60 min were needed with 2.75 mg Fe^2+^/L for achieving the SDS removal below the detection limit.

1 $$ {\mathrm{Fe}}^{2+}+{\mathrm{H}}_2{\mathrm{O}}_2\to {\mathrm{Fe}}^{3+}+{\mathrm{H}\mathrm{O}}^{-}+{\mathrm{H}\mathrm{O}}^{\bullet } $$

2 $$ {\mathrm{Fe}}^{3+}+{\mathrm{H}}_2\mathrm{O}+\mathrm{hv}\to {\mathrm{Fe}}^{2+}+{\mathrm{H}\mathrm{O}}^{\bullet }+{\mathrm{H}}^{+} $$

In the inset of Fig. 2a, the profiles of DOC mineralization are shown. It should be noted here that DOC corresponds to both the organic contaminants present in the synthetic wastewater (i.e., peptone and components of meat extract) and the model surfactant. Since the hydroxyl radicals are consumed in the oxidation of all the organic substances, the mineralization of SDS is affected by the presence of other organic contaminants. The DOC mineralization proceeded up to 30 min and 60 min with 10 and 5.5 mg Fe^2+^/L, respectively, stopping later due to the lack of hydrogen peroxide. In both cases the DOC content at the end of experiment amounted to ca. 10 mg/L; however, the initial stage of mineralization was very different. In the case of the highest concentration of ferrous iron a significant decrease of DOC content just at the beginning of the irradiation was found, while the changes observed for 5.5 mg Fe^2+^/L were significantly slower. That corresponds to the initial drop by Fenton process discussed above and confirms the importance of high iron concentration during this stage. At the lowest concentration of ferrous iron (2.75 mg Fe^2+^/L) the mineralization was not effective and the DOC content remained almost unchanged during 2 h of the process realization.

The evolution of hydrogen peroxide concentration with time is shown in Fig. 2b. At first glance, it can be seen that the effects observed for SDS degradation and DOC mineralization are well reflected in hydrogen peroxide consumption. When 5.5 and 10 mg Fe^2+^/L were used, the hydrogen peroxide consumption was > 99% after 60 and 40 min, respectively. This is consistent since H_2_O_2_ consumption rate is proportional to the concentration of the catalyst. Nevertheless, 50% H_2_O_2_ was still in the system at the end of the treatment with 2.75 mg Fe^2+^/L.

Moreover, based on the results presented in Fig. 2b it can be seen that in the case of 2.75 mg Fe^2+^/L the H_2_O_2_ consumption rate is very low (0.37 mg/L min). This is because at low ferrous iron content the radiation was utilized in a small degree, which resulted in a limited H_2_O_2_ consumption. However, for higher ferrous iron concentration when photons reaching reactor surface are fully absorbed, the H_2_O_2_ consumption rates are proportional to the Fe content, and reach 1.8 mg/L min for 5.5 mg Fe^2+^/L and 3.0 mg/L min for 10 mg Fe^2+^/L. Calculating the ratio of initial Fe^2+^ concentration (5.5/10 = 0.55) and the ratio of H_2_O_2_ consumption rate (1.8/3.0 = 0.6) in both experiments, it can be found that they are closely similar.

### Effect of H_2_O_2_ concentration on SDS removal

**Fig. 3:**
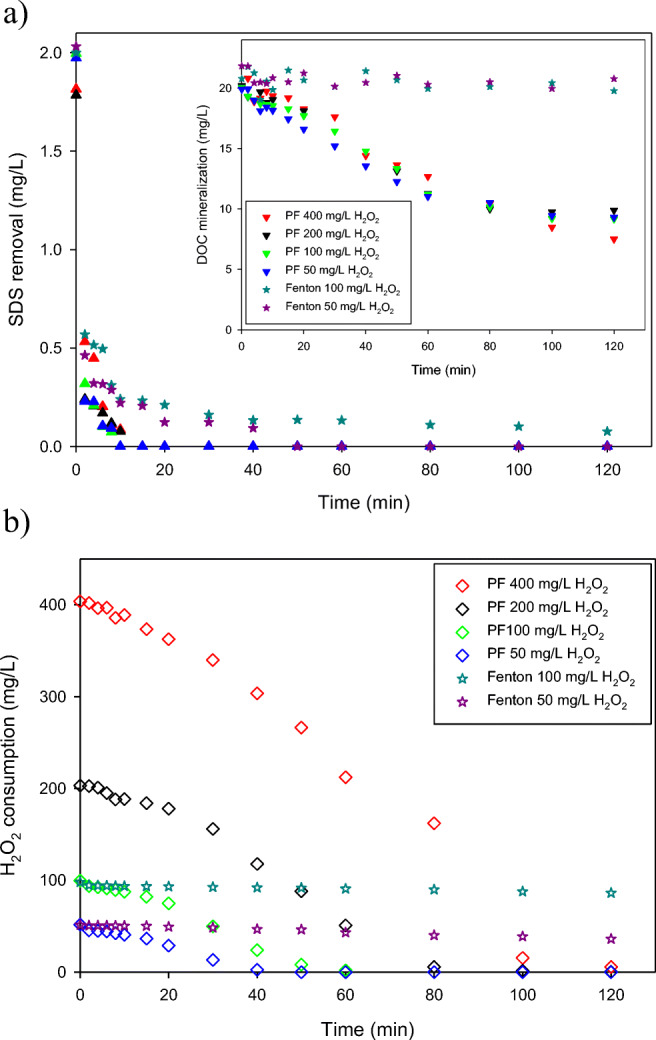
Fenton and solar photo-Fenton (PF) processes applied on 2 mg SDS/L with 5.5 mg Fe^2+^/L and four concentrations of hydrogen peroxide in the synthetic secondary effluent (SW). **a** Removal of SDS and DOC mineralization (inserted figure). **b** Hydrogen peroxide consumption

In order to study the effect of hydrogen peroxide concentration on SDS removal and DOC mineralization, a second experimental set was carried out using 5.5 mg/L of Fe^2+^. Such a concentration was selected because at both higher iron contents the SDS was almost completely removed in 10 min of the process. The effect of the Fenton reaction in these conditions was studied as a blank. The results show that SDS degradation rate by solar photo-Fenton is fast, reaching > 99% of removal with the four hydrogen peroxide concentrations in 15 min of the treatment time (Fig. 3a). The first step is always marked by a high and fast oxidation of the compounds due to the initial reaction of ferrous iron with hydrogen peroxide (Reaction (1)) (Fenton effect). This is reflected in the similar degree of SDS removal (Fig. 3a) for both the Fenton and the photo-Fenton processes at the very beginning of the experiments. However, subsequently, the decomposition of SDS proceeded significantly faster during the processes involving UV irradiation, even for the lowest dose of hydrogen peroxide (i.e., 50 mg/L). Hydroxyl radicals formed under the UV radiation took part in the direct degradation of SDS and the oxidation of other organic compounds present in the wastewater (such as peptone or components of meat extract, and by-products formed during decomposition), as well as in the redox cycle with the participation of Fe^3+^ ions. Hence, a more effective SDS removal and DOC degradation after 120 min of experiment took place in the case of the PF, achieving approximately 50% of DOC mineralization for H_2_O_2_ doses in the range of 50–200 mg/L, and in the case of the highest concentration of H_2_O_2_ (i.e., 400 mg/L) still some low progress of DOC removal was observed. Regarding the DOC profiles during the dark Fenton process, no noticeable changes of the concentration were found in both cases, demonstrating that the increase of H_2_O_2_ dose had no positive influence on the improvement of the mineralization (inset of Fig. 3a). These results revealed that the efficiency of the Fenton process in laundry wastewater treatment is significantly lower compared with that of the photo-Fenton system. This is because in the Fenton process the hydroxyl radicals are formed only during the oxidation of Fe^2+^ to Fe^3+^ represented by Reaction (1), while during the solar photo-Fenton process a continuous generation of HO^·^ proceeds (Reaction (2)).

In Fig. 3b, the curves representing H_2_O_2_ consumption with time are compared. In general, the consumption of this oxidant can be adjusted to the pseudo-first order kinetics. This can be observed in Fig. 3b, when 400 mg H_2_O_2_/L are used the consumption rate is 3.6 mg/L min, while with 50 mg/L it is 1.2 mg/L min. Hydrogen peroxide was completely consumed in the system after 120 min of the treatment only in the case of the initial concentration equal to 50 and 100 mg H_2_O_2_/L, while at the highest dose of the oxidant the final value was about 5 mg/L.

In the Fenton process, the slower regeneration of ferrous iron in the absence of light resulted in an insignificant hydrogen peroxide consumption (Fig. 3b). Thus, fewer hydroxyl radicals were yielded and DOC concentration did not change during the process (inset of Fig. 3a). Nevertheless, the small amounts of radicals generated were enough to oxidize the SDS, but more slowly than in the solar photo-Fenton process (Fig. 3a).

### Effect of the aqueous matrix composition on SDS removal

**Fig. 4:**
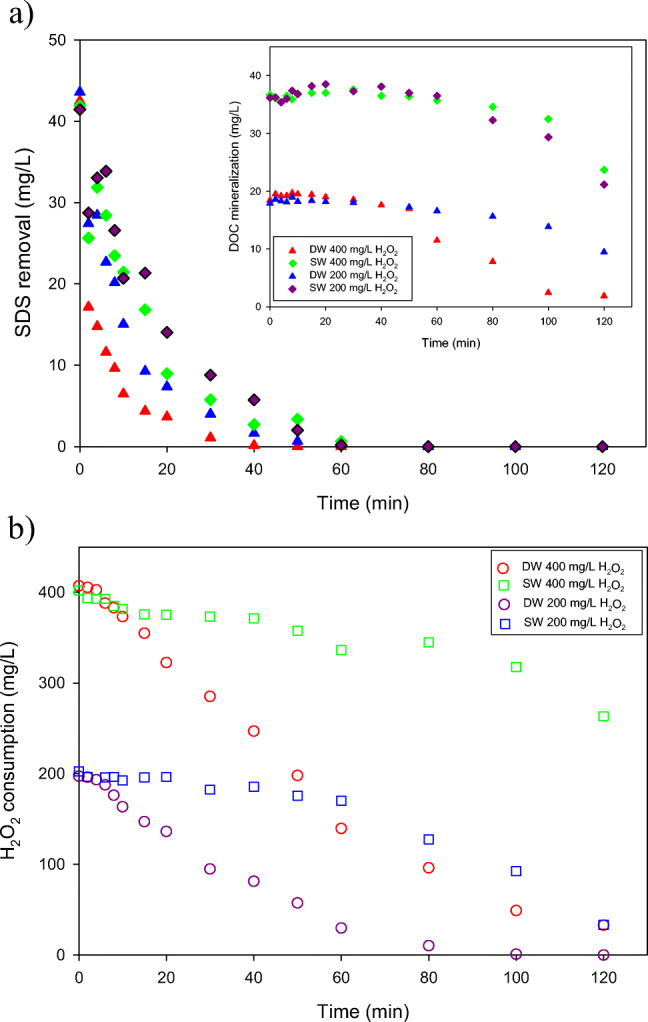
Effect of solar photo-Fenton process on removal of 40 mg SDS/L with 5.5 mg Fe^2+^/L and two concentrations of hydrogen peroxide: 400 mg/L and 200 mg/L, using distilled water (DW) and synthetic secondary effluent (SW). **a** SDS removal and DOC mineralization (inserted figure) of 40 mg SDS/L. **b** Consumption of hydrogen peroxide

In view of the above results and to demonstrate that the SDS is mineralized, different experiments were carried out in two matrices, synthetic wastewater (SW) and distilled water (DW) with the highest concentrations of hydrogen peroxide, 400 and 200 mg/L and SDS concentration increased to 40 mg/L, to take into account the effect of organic matter concentration on the photo-Fenton process performance (Ballesteros Martín et al. 2009). As can be observed in Fig. 4a, the SDS oxidation was not influenced by the concentration of H_2_O_2_ in SW obtaining the same SDS degradation, > 99%, in 60 min of the treatment with both hydrogen peroxide concentrations. However, the treatment time to remove SDS below the detection limit is shorter in DW (40 min with 400 mg H_2_O_2_/L) than in SW (60 min) due to the lack of the additional organic matter. Accordingly, the hydroxyl radicals generated during the photo-Fenton process attacked the model surfactant solely. The DOC mineralization is shown in the inset of Fig. 4a. During the first 60 min, the SDS is mainly oxidized giving place to an initial shoulder in the DOC removal profile. The process of DOC mineralization begins when a complete removal of SDS is reached, regardless of the applied matrix. In the case of SW, the mineralization percentage is similar with both hydrogen peroxide concentrations (around 40%), whereas this percentage increases with 400 mg H_2_O_2_/L in DW achieving the total mineralization below the detection limit in 120 min of the treatment process. A non-substantial difference between DOC mineralization profiles in SW for the two H_2_O_2_ concentrations (inset in Fig. 4a) can be attributed to the presence of various and complex organic compounds originating from peptone and meat extract (Cabrera Reina et al. 2018). These substances could hinder the DOC mineralization, even for such high H_2_O_2_ concentration. This is because of the non-selective character of hydroxyl radicals, which can react with various organic species. In case of DW, only a decomposition of high amount of SDS and its further mineralization took place.

Based on the changes of H_2_O_2_ concentration in DW and SW presented in Fig. 4b, it can be found that the consumption of H_2_O_2_ in DW was greater than in SW, which corresponds well with the DOC mineralization profile (Fig. 4a). The rates of H_2_O_2_ removal in DW amounted to 2.9 and 3.9 mg/L min for 200 and 400 mgH_2_O_2_/L, respectively, while in the case of SW they were equal to 0.5 and 0.7 mg/L min only. It is also worth noting that the consumption of hydrogen peroxide in DW was similar to that observed in the experiments presented in Fig. 3, in which the initial DOC concentration was comparable (about 20 mg/L). In case of 200 mgH_2_O_2_/L the consumption rates were 2.9 and 2.5 mg/L min, respectively, while for 400 mgH_2_O_2_/L they amounted to 3.6 and 3.9 mg/L min, respectively. These results indicate that the type of organic contaminants (i.e., SDS solely (Fig. 4) or SDS in SW (Fig. 3)) was not important, confirming the non-selective character of hydroxyl radicals participating in the mineralization process.

### A comparison of SDS removal under acidic and neutral pH

The main disadvantage of the photo-Fenton process is a need of acidification of the reaction medium (pH < 3) in order to operate the system with high efficiency. To overcome this drawback the application of EDDS under neutral pH can be considered. This chemical is biodegradable and safe for the environment; therefore, its utilization as the iron complexing agent for the PF process could be a practical solution (Arzate et al. 2020; Hinojosa Guerra et al. 2019). Hence, assays at neutral pH using Fe^3+^-EDDS complex were additionally carried out. To easy compare the results, the selected process conditions reflected those in the first set of experiments (Fig. 2), i.e., concentration of hydrogen peroxide was 100 mg/L, while that of SDS amounted to 2 mg/L. The Fe^3+^:EDDS molar ratio equal to 1:1 was selected on a basis of literature data. For example, Arzate et al. (2020) reported that the removal of contaminants of emerging concern from secondary effluents of municipal wastewater treatment plant through the solar PF at neutral pH was more efficient at 1:1 molar ratio of Fe^3+^:EDDS and hydraulic residence time (HRT) of 20 min than at 1:2 ratio and HRT of 40 min.

**Fig. 5:**
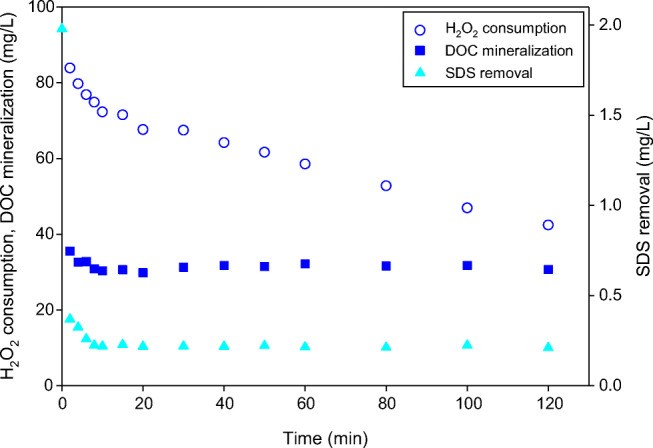
Solar PF at neutral pH for 2 mg SDS/L in synthetic secondary effluent (SW) with 100 mg H_2_O_2_/L and 5.5 mg/L of Fe^3+^:EDDS

As can be observed in Fig. 5, the solar photo-Fenton was able to remove ca. 89% of SDS and mineralize 13% of DOC during first 8 min, corresponding to the time in which the complex was present in the solution. Later, due to Fe^3+^-EDDS photodecomposition, the degradation stopped and the values of both parameters remained unchanged up to the end of the process. The presence of Fe^3+^-EDDS contributed to a higher rate of H_2_O_2_ consumption (1.50 mg/L min) during the lifetime of the complex (less than 10 min) compared with the second stage of the process. The decomposition of the complex caused a decrease in the H_2_O_2_ consumption rate to 0.27 mg/L min (Fig. 5). The residual H_2_O_2_ concentration was very high and amounted to 40 mg/L. The obtained results revealed the possibility of SDS decomposition; however, the DOC mineralization was on a very low level. Nonetheless, opposite to the conventional photo-Fenton, the complete SDS removal was not achieved (Fig. 5). In the case of PF process, even for the lowest hydrogen peroxide concentration, the SDS removal below the detection limit was noted after 8 min (Fig. 3a). Also, mineralization of DOC was more efficient in PF. For the same H_2_O_2_ dose (100 mg/L) it systematically proceeded through 80 min, and after 120 min of the process it exceeded 50% (Fig. 3a).

Despite a relatively high rate of SDS decomposition, a disadvantage of the application of the Fe^3+^-EDDS complex is a high concentration of organic load in the solution resulting from EDDS addition. In case of utilization of this type of AOP for post-treatment of laundry wastewater aimed at its reuse in the laundry cycle, an additional treatment step aimed at the removal of the organic contaminants must be considered.

## Conclusions

The removal of the anionic surfactant, sodium dodecyl sulphate, from a synthetic secondary effluent of a laundry wastewater treatment system in a pilot scale solar photo-Fenton plant was investigated. The results indicated that when the initial surfactant concentration was 2 mg/L the most beneficial PF operational conditions under solar light were 5.5 mgFe^2+^/L and 100 mgH_2_O_2_/L. For these parameters the SDS degradation exceeded 99% in 15 min and the DOC mineralization reached about 50% in 120 min of irradiation. The investigations on the influence of the aqueous matrix composition revealed that decomposition of 40 mg/L SDS in distilled water was faster than in the synthetic wastewater, although in both cases the concentration of the surfactant decreased below the detection limit in 60 min or less. On the opposite, DOC mineralization was significantly less effective in the case of the sewage due to the presence of other organic contaminants competing with SDS for the hydroxyl radicals. The DOC mineralization at 5.5 mgFe^2+^/L and 400 mgH_2_O_2_/L reached almost 100% when distilled water was used as a matrix and only about 40% in the case of the synthetic sewage. The consumption of H_2_O_2_ was greater in DW compared with SW and corresponded well with changes of DOC concentration. Moreover, it was found that the H_2_O_2_ consumption rates in various solutions containing similar DOC amount but of different origin (i.e., associated solely with SDS dissolved in DW or related to various organic contaminants in the SW) were similar, which confirmed the non-selective character of hydroxyl radicals participating in the mineralization process.

The analysis of SDS removal under dark Fenton conditions revealed that this process is inefficient in laundry wastewater treatment. Moreover, for comparison purpose the treatment of the synthetic wastewater was also realized at neutral pH with application of EDDS as the iron complexing agent. The applied conditions allowed to decompose SDS with good efficiency (89% in 8 min), although slightly lower than under acidic conditions (96%). However, a disadvantage is that the effluent still needs post-treatment due to the presence of high DOC content arising from EDDS addition, which excludes it from direct reuse in the laundry process.

The experiments revealed a great potential of the solar photo-Fenton realized at acidic pH in terms of post-treatment of biologically pre-treated laundry wastewater. Future work should be focused on finding the conditions for improved mineralization of DOC and removal of iron in order to create a possibility of recycling of the post-treated effluent to the laundry process.

## Data Availability

The datasets used and/or analysed during the current study are available from the corresponding author on reasonable request.

## List of symbols and abbreviations

AOPs: Advanced oxidation processes
CPC: Compound parabolic collector
DOC: Dissolved organic carbon
DW: Distilled water
EDDS: Ethylenediamine-N,N’-disuccinic acid
F: Fenton
HPLC-DAD: High-performance liquid chromatography with diode array detector
IC: Inorganic carbon
LOQ: Limit of quantification
PF: Photo-Fenton
SDS: Sodium dodecyl sulphate
SW: Synthetic wastewater
TOC: Total organic carbon
UV: Ultraviolet
*V*_*i*_: Illuminated volume
*V*_*T*_: Total volume
